# Papillary Fibroelastoma of the Aortic Root Causing Intermittent Coronary Ostial Obstruction: The Diagnostic Power of 3D Transesophageal Echocardiography

**DOI:** 10.3390/diagnostics16010168

**Published:** 2026-01-05

**Authors:** Tina Bečić, Ružica Perković-Avelini, Damir Fabijanić

**Affiliations:** 1 Department of Cardiovascular Diseases, University Hospital of Split, 21000 Split, Croatia; tina.becic@gmail.com (T.B.); ruzica.perkovic.avelini@gmail.com (R.P.-A.); 2 Clinical Skills Department III, University of Split School of Medicine, 21000 Split, Croatia

**Keywords:** papillary fibroelastoma, tumor, heart, aorta, chest pain, echocardiography

## Abstract

We describe a patient with recurrent, brief episodes of chest discomfort caused by a highly mobile papillary fibroelastoma originating from the aortic wall and intermittently encroaching on the right coronary artery ostium. Initial 2D and 3D transthoracic and 2D transesophageal echocardiography identified a highly mobile mass in the ascending aorta above the aortic valve; the exact site of attachment and its relationship to the coronary ostia could not be clearly defined. Three-dimensional transesophageal echocardiography enabled precise anatomical reconstruction of the lesion and surrounding structures, clearly demonstrating its pedicle and proximity to the right coronary ostium. This imaging modality clarified the pathophysiological mechanism of symptoms and facilitated optimal surgical planning without the need for additional complex imaging techniques.

**Figure 1:**
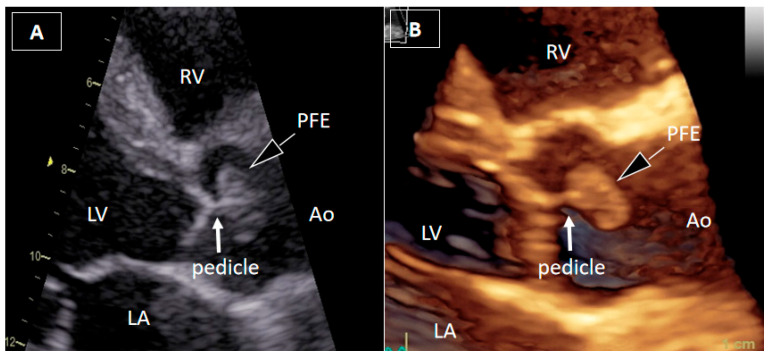
Transthoracic echocardiography. A 54-year-old man with recurrent, brief episodes of chest discomfort and no cardiovascular risk factors was referred for echocardiographic evaluation. Two-dimensional transthoracic echocardiography (2DTTE) (**A**) and three-dimensional transthoracic echocardiography (3DTTE) (**B**) demonstrated a round, mobile mass in the ascending aorta just above the aortic valve, most likely attached to the downstream surface of the right valve leaflet by a short pedicle. The attachment site of the pedicle was not clearly identified. Other structural and functional echocardiographic parameters, including myocardial strain imaging, were within normal ranges.

**Figure 2:**
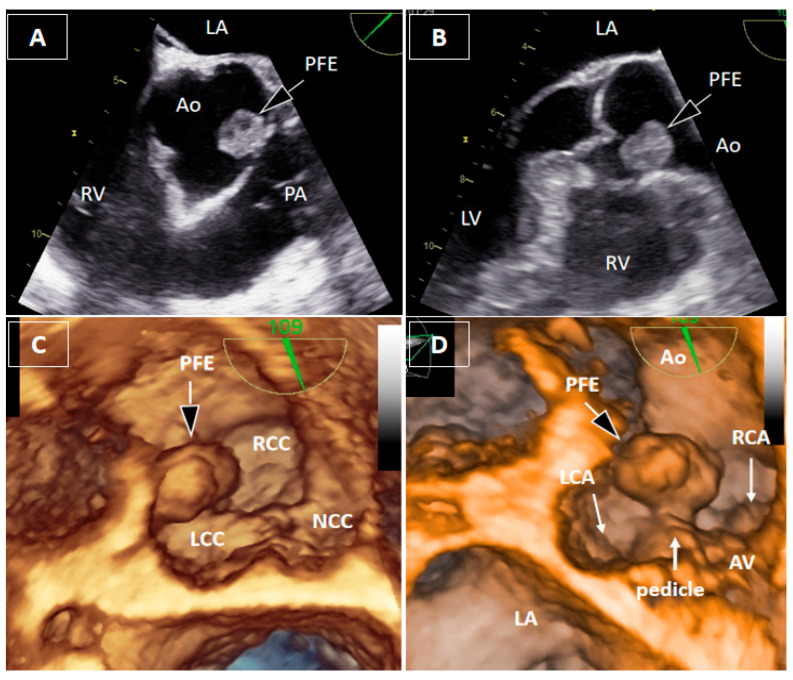
Transesophageal echocardiography. Two-dimensional transesophageal echocardiography (2DTEE) localized the mass to the region of the right coronary sinus, between the left and right coronary cusps, without precise identification of the attachment site (**A**,**B**). Three-dimensional transesophageal echocardiography (3DTEE) provided detailed anatomical definition, clearly identifying a short pedicle originating from the aortic wall between the bases of the left and right coronary cusps (**C**,**D**). The mass measured 17 × 11 × 8 mm, with a pedicle length of approximately 4 mm, and demonstrated marked mobility toward the right coronary artery ostium during the cardiac cycle. Measurements were obtained from the 3DTEE.

**Figure 3:**
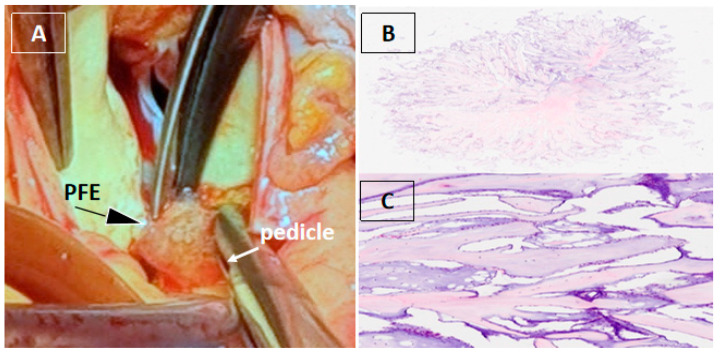
Surgical and histopathological findings. Surgical removal of a cardiac mass is recommended in both symptomatic and asymptomatic patients due to the risk of embolization, especially when the mass is mobile and larger than 10 mm [1,2,3]. Given the patient’s symptoms, the marked mobility of the lesion, and its immediate proximity to the right coronary ostium with suspected dynamic obstruction and high embolic risk, surgical excision was performed. Computed tomography coronary angiography, performed to rule out occult coronary disease as an alternative cause of chest discomfort, showed coronary arteries without atherosclerotic disease [4,5]. Cardiac magnetic resonance imaging can be useful for tissue characterization and defining the anatomical relationship of the mass to adjacent structures [3,4,5,6]. However, in our patient, 3DTEE provided sufficient anatomical definition of the mass and its relationship to the coronary ostium, obviating the need for additional imaging. Intraoperative inspection revealed a gelatinous mass attached by a small pedicle to the aortic wall immediately adjacent to the right coronary artery ostium, which was completely excised with preservation of the aortic valve and root (**A**). Histopathological analysis confirmed papillary fibroelastoma (PFE), characterized by avascular papillary fronds with a fibroelastic core lined by endothelium (**B**,**C**) [7].

**Figure 4:**
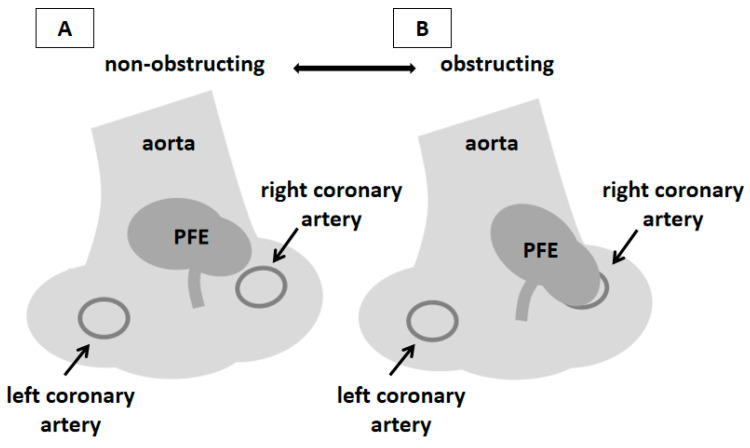
Schematic representation of the localization of PFE and the pathophysiological mechanism of the patient’s symptoms. PFE typically arises from the downstream surface of left-sided valves, most frequently aortic; localization in the aortic wall is extremely rare [6,8,9]. In the presented patient, the onset of symptoms was closely related to the mobility of the mass and its occasional contact with the ostium of the right coronary artery. Similar cases have already been described in the literature [10,11,12,13,14]. 3DTEE enabled direct visualization of this interaction, providing pathophysiological insight and facilitating tailored surgical planning. This schematic illustration (prepared from Figure 2D) summarizes the hypothesized mechanism of intermittent ostial obstruction of the right coronary artery. During the cardiac cycle, a mobile PFE arising from the aortic wall adjacent to the right coronary artery ostium moves toward and away from the ostium, occasionally resulting in its transient occlusion and consequent myocardial ischemia (**A**,**B**). During the four-year follow-up, the patient remained asymptomatic with normal echocardiographic findings. Therefore, the resolution of symptoms after the PFE excision points to this mechanism as the most likely cause of the symptoms in our patient.

## Data Availability

The original contributions presented in this study are included in the article. Further inquiries can be directed to the corresponding author.

## Abbreviations

The following abbreviations are used in this manuscript:

Ao	Aorta
AV	Aortic valve
LA	Left atrium
LCA	Left coronary artery
LCC	Left coronary cusp
LV	Left ventricle
NCC	Non coronary cusp
PFE	Papillary fibroelastoma
PA	Pulmonary artery
RCA	Right coronary artery
RCC	Right coronary cusp
RV	Right ventricle
2DTTE	Two-dimensional transthoracic echocardiography
3DTTE	Three-dimensional transthoracic echocardiography
2DTEE	Two-dimensional transesophageal echocardiography
3DTEE	Three-dimensional transesophageal echocardiography
